# The Carotid–Hyoid Topography Is Variable

**DOI:** 10.3390/medicina59081494

**Published:** 2023-08-19

**Authors:** Mihaela Daniela Manta, Mugurel Constantin Rusu, Sorin Hostiuc, Alexandra Diana Vrapciu, Bogdan Adrian Manta, Adelina Maria Jianu

**Affiliations:** 1 Department of Anatomy and Embriology, Faculty of Medicine, Victor Babeș University of Medicine and Pharmacy, 300041 Timișoara, Romania; monea.mihaela@umft.ro (M.D.M.); adelina.jianu@umft.ro (A.M.J.); 2 Division of Anatomy, Faculty of Dentistry, Carol Davila University of Medicine and Pharmacy, 050474 Bucharest, Romania; alexandra.vrapciu@umfcd.ro; 3 Division of Legal Medicine and Bioethics, Faculty of Dentistry, Carol Davila University of Medicine and Pharmacy, 050474 Bucharest, Romania; sorin.hostiuc@umfcd.ro; 4 Division of Clinical Practical Skills, Faculty of Medicine, Victor Babeș University of Medicine and Pharmacy, 300041 Timișoara, Romania; manta.bogdan@umft.ro

**Keywords:** carotid artery, carotid bifurcation, computed tomography, larynx, pharynx

## Abstract

*Background and Objectives*: The carotid bifurcation (CB) is presented in most anatomy textbooks as having a unique location at the upper margin of the thyroid cartilage. Although a number of case reports have provided evidence of the possibility of carotid artery location either lateral or medial to the greater hyoid horn, these reports have not established specific anatomic possibilities and prevalences. *Materials and Methods*: We retrospectively analysed a batch of 147 CT angiograms for 12 types of carotid–hyoid relationships and classified the bilateral combination possibilities of these types. *Results*: In 168/294 sides there were no carotid–hyoid relationships. Type I, external carotid artery (ECA) medial to the greater horn of the hyoid bone (GHHB), was observed in 0.34%; type II, internal carotid artery (ICA) medial to GHHB, in 0.34%; type III, ICA and ECA medial to GHHB, in 1.02%; type IV, common carotid artery (CCA) medial to GHHB, in 1.02%; type V, CB medial to GHHB, in 0.34%; type VI, ECA lateral to GHHB, in 20.41%; type VII, ICA lateral to GHHB, was not recorded; type VIII, ECA and ICA lateral to GHHB, in 3.74%; type IX, CCA lateral to GHHB, in 8.5%; type X, CB lateral to GHHB, in 6.46%; type XI, ECA lateral and ICA medial to GHHB, in 0.34%; and type XII, ICA lateral and ECA medial to GHHB, in 0.34%. Bilateral symmetry was found in 70.74% of cases, including the null types without carotid–hyoid relationships as well as types IV, VI, VIII, IX, and X. There was a highly significant association between the left and right variants of the carotid–hyoid relationship. *Conclusions*: Mechanical compression of the hyoid bone on the carotid arteries has various undesirable effects on the ICA and cerebral circulation. Underlying these are several variational anatomical patterns of carotid–hyoid relationships, which can be accurately documented on CT angiograms. A case-by-case anatomical study is better than assuming the carotid anatomy learned from textbooks.

## 1. Introduction

Usual anatomical descriptions indicate that the common carotid artery (CCA) normally bifurcates at the superior margin of the thyroid cartilage into the external (ECA) and internal (ICA) carotid arteries [1,2,3]. The superior margin of the thyroid cartilage is, however, just the mean level of CB [4]. During the developmental stages, the geometry of the CB changes considerably; at the beginning, the ICA appears to emanate as a side branch, while the ECA appears to be a continuation of the CCA, while later both daughter vessels represent a skewed continuation of the parent artery [5]. The carotid bifurcation (CB) is not typically indicated with a variable vertical location referring to the thyroid cartilage or the greater horn of the hyoid bone (GHHB), nor are the possible variable relations between the carotid arteries and the GHHB commonly considered. For example, Gray’s Anatomy describes the CB as usually being located “above the level of the thyroid lamina” [6]; however, this neither includes, nor excludes direct carotid–hyoid relationships. As observed previously, human anatomy texts in current use have very little precise information as to the frequency of variations in the CB [7]. The carotid–hyoid relationships should, however, have a certain degree of variability in time, as different studies have assessed that the transverse topography of carotid arteries and their interrelationships could vary [8,9,10,11,12,13].

Therefore, in this paper we aimed to detail the possible carotid–hyoid relationships and their occurrence.

## 2. Materials and Methods

There were 150 archived consecutive angioCT scans performed in the period November 2022–April 2023. Inclusion criteria were good quality of scans (motion-free, no background noise, adequate vascular enhancement, lack of image artefacts), adequate vertical height of scans, and no pathological processes distorting the carotid anatomy. Exclusion criteria were scans inadequate for observing the carotid arteries and pathological processes nearing the carotid arteries and distorting their anatomical features. On this basis, we excluded three cases. Thus, determinations were made on a retrospectively assessed batch of 147 cases, including 86 males and 61 females (sex ratio = 1.4).

All subjects provided their informed consent for inclusion before participating in the study. The research was conducted following principles from the Code of Ethics of the World Medical Association (Declaration of Helsinki). The responsible authorities (Affiliation 1) approved the study (approval no. 45/4 September 2020).

The CTAs were performed with a 32-slice scanner (Siemens Multislice Perspective Scanner, Forcheim, Germany) with a 0.6 mm collimation and a reconstruction of 0.75 mm thickness with 50% overlap for a multiplanar maximum intensity projection and the three-dimensional volume rendering technique, as described previously [14]. The cases were documented using Horos for iOS (Horos Project), as in previous studies [15]. Findings were checked on two-dimensional planar reconstructions and documented with three-dimensional volume renderings.

To assess the variability of carotid–hyoid topographical relations, twelve variational possibilities (types) of the interrelations between the carotid artery and greater horn of the hyoid bone (GHHB) were defined: (1) type I—ECA medial to GHHB; (2) type II—ICA medial to GHHB; (3) type III—both ECA and ICA medial to GHHB; (4) type IV—CCA medial to GHHB; (5) type V—CB medial to GHHB; (6) type VI—ECA lateral to GHHB; (7) type VII—ICA lateral to GHHB; (8) type VIII—both ECA and ICA lateral to GHHB; (9) type IX—CCA lateral to GHHB; (10) Type X—CB lateral to GHHB; (11) Type XI—ICA medial to greater horn, ECA lateral to it; and (12) Type XII—ECA medial to greater horn, ICA lateral to it. Cases in which the carotid arteries were posterior to the tip of the GHHP, meaning that no direct carotid–hyoid relation was recorded, were regarded as null types.

To assess the statistical significance of the bilateral symmetry of types, we used the Pearson Chi-squared test. A *p*-value below 0.05 was considered significant.

## 3. Results

**Figure 1:**
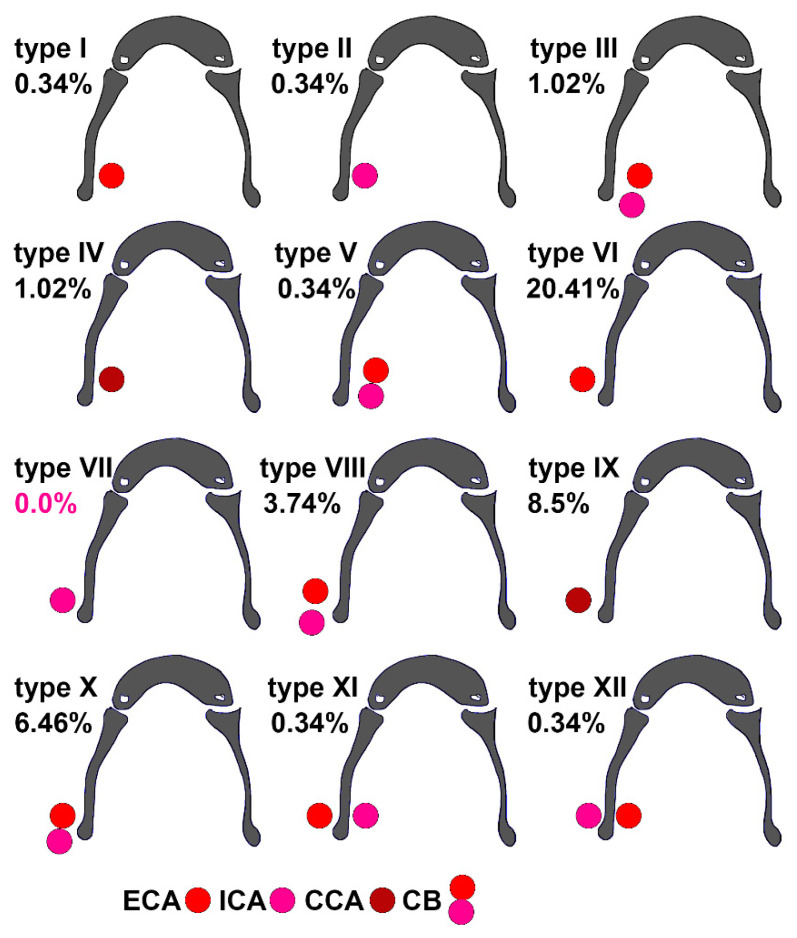
Prevalence and diagrams of types I–XII of the carotid–hyoid relationship in the general lot (147 cases, 294 sides). ECA: external carotid artery; ICA: internal carotid artery; CCA: common carotid artery; CB: carotid bifurcation.

In 57.14% of cases, no direct carotid-hyoid anatomical relations (types I–XII) were identified in the overall batch bilaterally (N = 294 CBs). In 42.86%, we found different types of carotid–hyoid anatomical relations, with the exception of type VII—ICA lateral to the GHHB (Figure 1).

**Figure 2:**
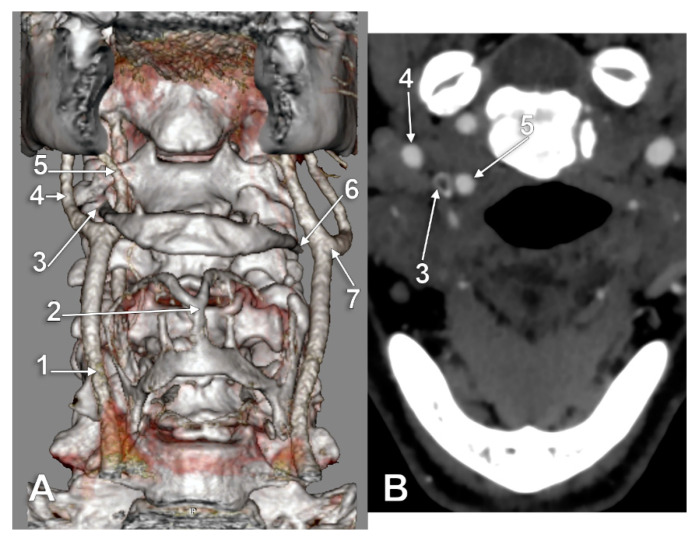
Type I carotid-hyoid relationship (right side), contralaterally tilted hyoid. Three-dimensional volume rendering, anterior view (**A**) and axial slice through tip of right greater hyoid horn (**B**). 1: right common carotid artery; 2: thyroid cartilage; 3: tip of right greater hyoid horn; 4: right internal carotid artery; 5: right external carotid artery; 6: tip of left greater hyoid horn; 7: left carotid bifurcation.

**Figure 3:**
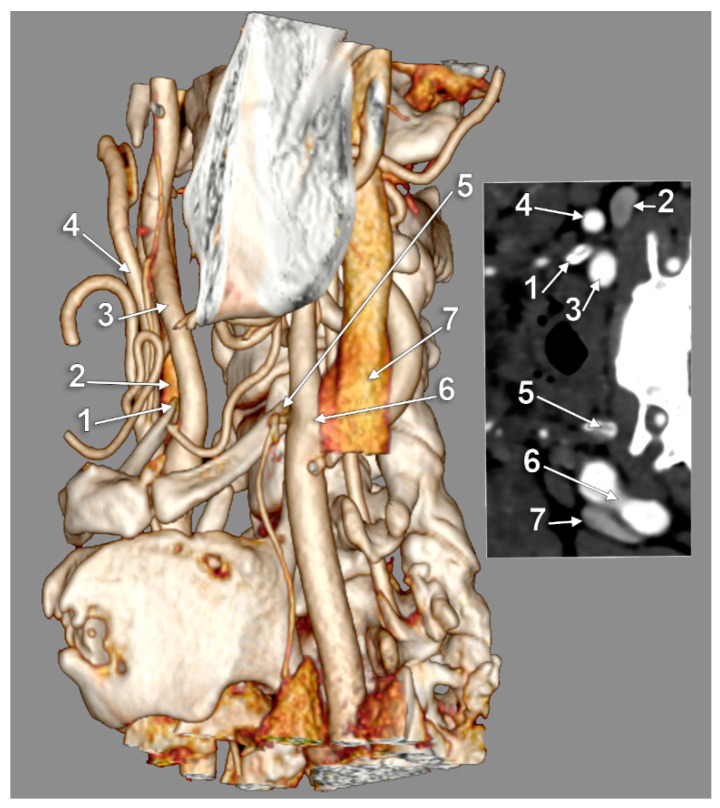
Bilateral asymmetric carotid–hyoid relationships: right type II and left type X. Three-dimensional volume rendering, left lateral view. Inset: axial slice through the tips of the greater hyoid horns. 1: tip of right greater hyoid horn; 2: right internal jugular vein; 3: right internal carotid artery; 4: right external carotid artery; 5: tip of left greater hyoid horn; 6: left carotid bifurcation; 7: left internal jugular vein.

**Figure 4:**
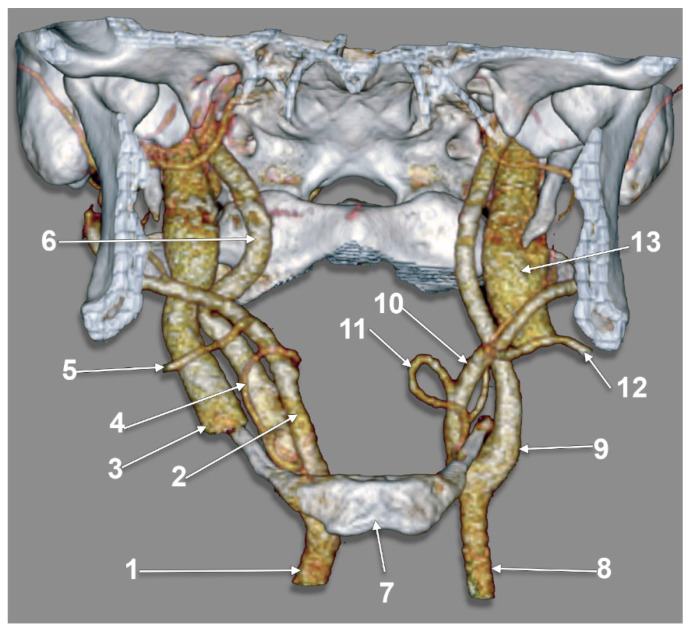
Types III (right side) and XII (left side) of carotid–hyoid relationships. Three-dimensional volume rendering, anterior view. 1: right common carotid artery; 2: right external carotid artery; 3: right internal jugular vein; 4: right lingual artery; 5: right facial artery; 6: right internal carotid artery; 7: body of hyoid bone; 8: left common carotid artery; 9: left internal carotid artery; 10: left external carotid artery; 11: left lingual artery; 12: left facial artery; 13: left internal jugular vein.

**Figure 5:**
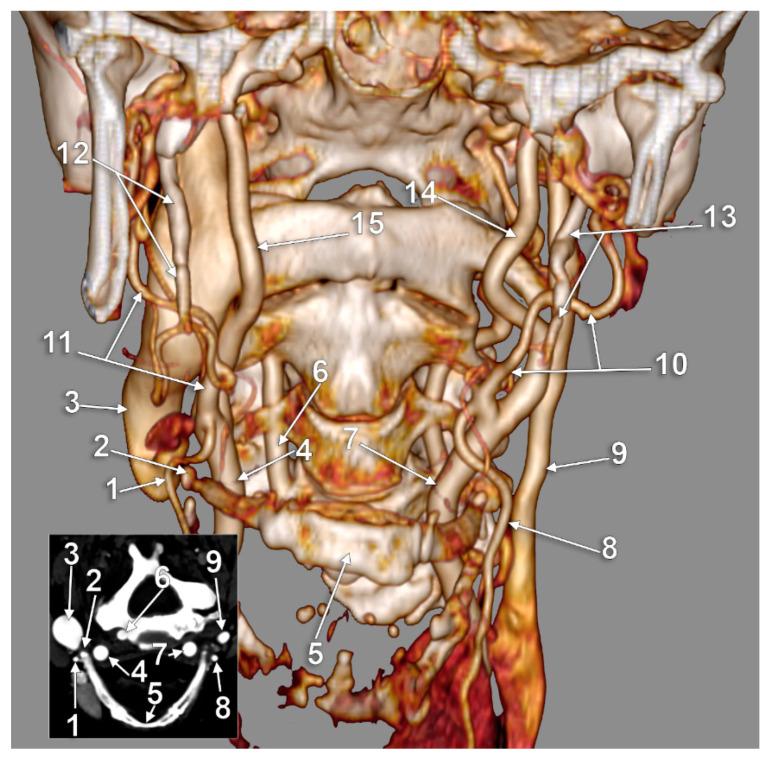
Bilateral type IV of carotid–hyoid relationship. The external carotid arteries cross the posteriorly elongated styloid processes. Three-dimensional volume rendering, anterior view. Inset: axial slice through hyoid. 1: right superior thyroid artery; 2: apex of right greater hyoid horn; 3: right internal jugular vein; 4: right common carotid artery; 5: body of hyoid; 6: right vertebral artery; 7: left common carotid artery; 8: left superior thyroid artery; 9: left internal jugular vein; 10: left external carotid artery; 11: right external carotid artery; 12: elongated right styloid process; 13: elongated left styloid process; 14: left internal carotid artery; 15: right internal carotid artery.

**Figure 6:**
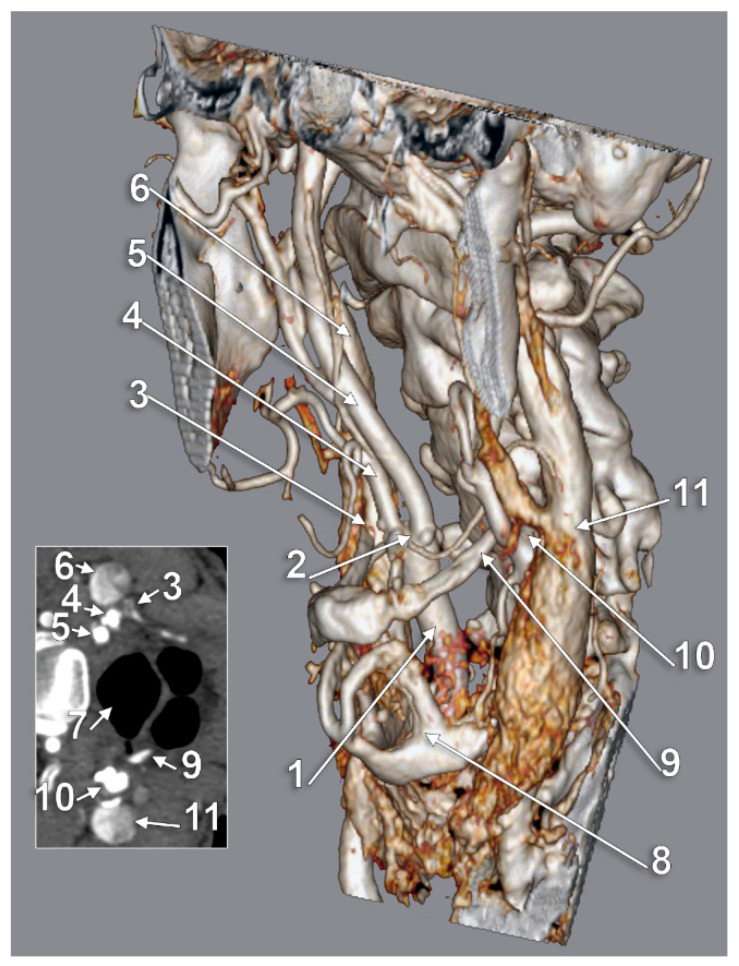
Bilateral combination types V and X of carotid–hyoid relationships. Three-dimensional rendering, left anterolateral view. Inset: axial slice through the greater hyoid horns. 1: right common carotid artery; 2: right carotid bifurcation; 3: tip of right greater hyoid horn; 4: right internal carotid artery; 5: right internal carotid artery; 6: right internal jugular vein; 7: pharynx; 8: thyroid cartilage; 9: left greater hyoid horn; 10: left carotid bifurcation; 11: left internal jugular vein.

**Figure 7:**
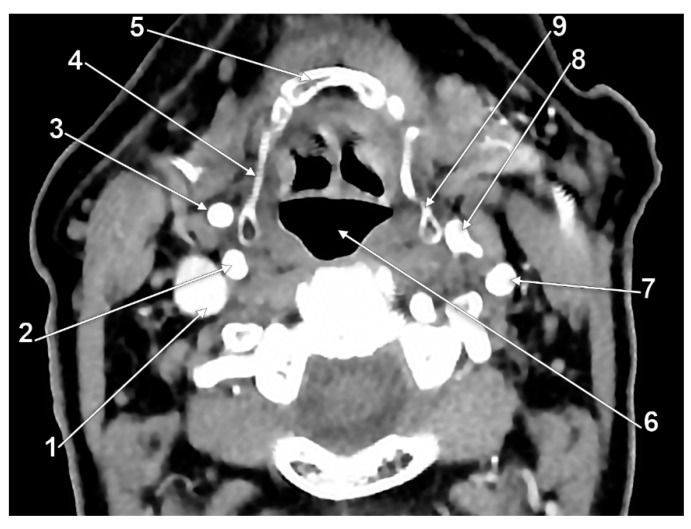
Bilateral type VI of carotid–hyoid relationship. Axial slice through the tips of the greater horns of the hyoid bone, inferior view. 1: right internal jugular vein; 2: right internal carotid artery; 3: right external carotid artery; 4: right greater horn of hyoid bone; 5: body of hyoid; 6: pharynx; 7: left internal carotid artery; 8: left external carotid artery; 9: left greater horn of hyoid bone.

**Figure 8:**
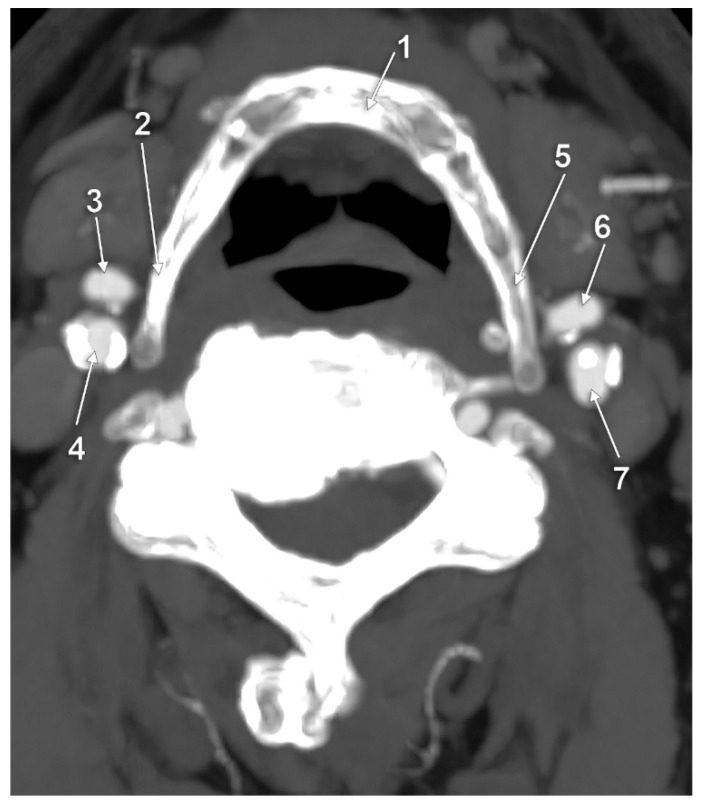
Bilateral type VII of carotid–hyoid relationship. Axial slice through the tips of the greater horns of the hyoid bone, inferior view. 1: body of hyoid bone; 2: right greater horn of hyoid bone; 3: right external carotid artery; 4: right internal carotid artery; 5: left greater horn of hyoid bone; 6: left external carotid artery; 7: left internal carotid artery.

**Figure 9:**
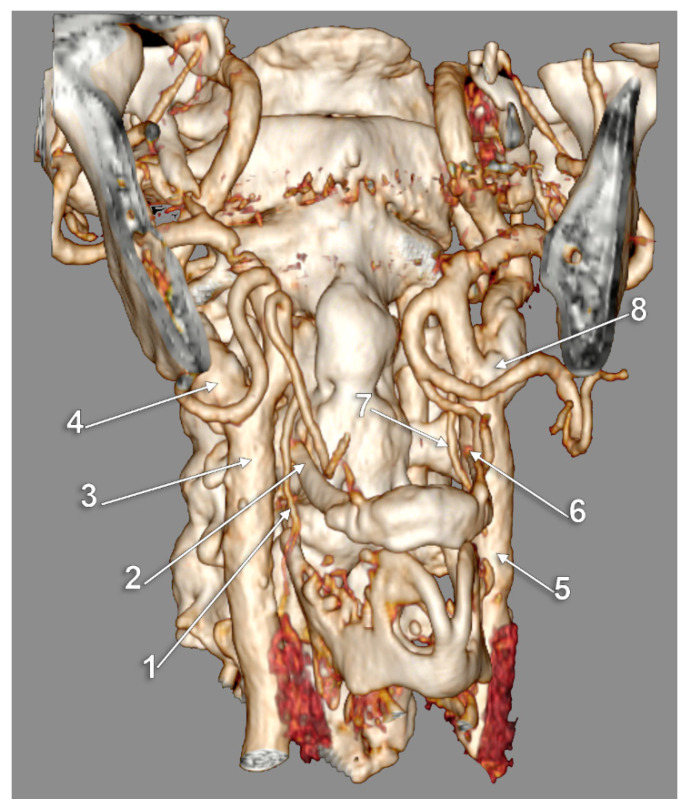
Bilateral type IX of carotid–hyoid relationship. Three-dimensional rendering, right antero-lateral view. 1: right superior thyroid artery; 2: right greater hyoid horn; 3: right common carotid artery; 4: right internal carotid artery; 5: left common carotid artery; 6: tip of left greater hyoid horn; 7: left superior thyroid artery; 8: left carotid bifurcation.

**Figure 10:**
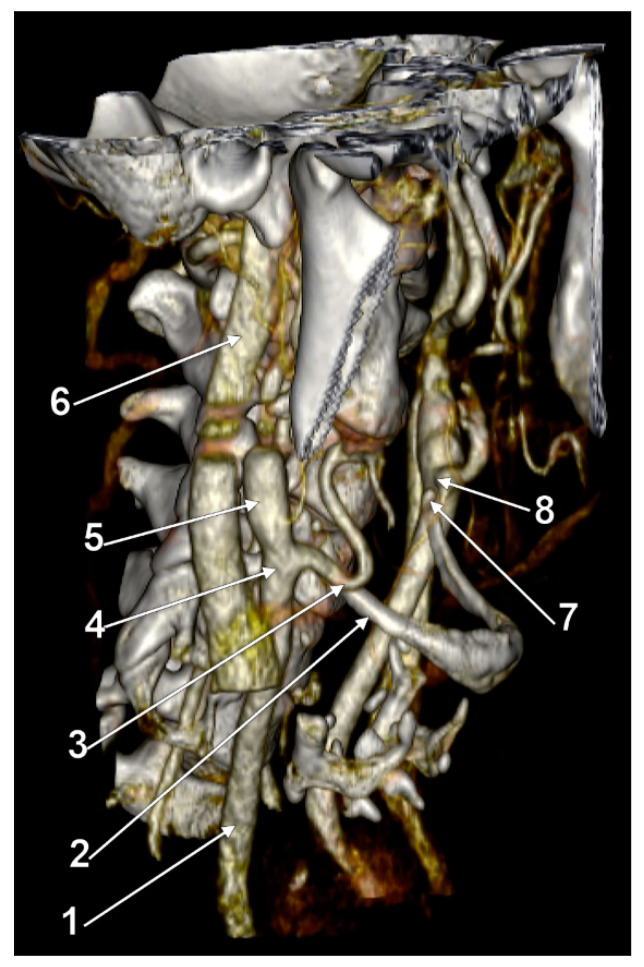
Bilateral type X of carotid–hyoid relationship. Hyoid loop of the right external carotid artery. Three-dimensional rendering, right anterolateral view. 1: right common carotid artery; 2: right greater hyoid horn; 3: right external carotid artery; 4: right carotid bifurcation; 5: right internal carotid artery; 6: right internal jugular vein; 7: tip of left greater hyoid horn; 8: left carotid bifurcation.

**Figure 11:**
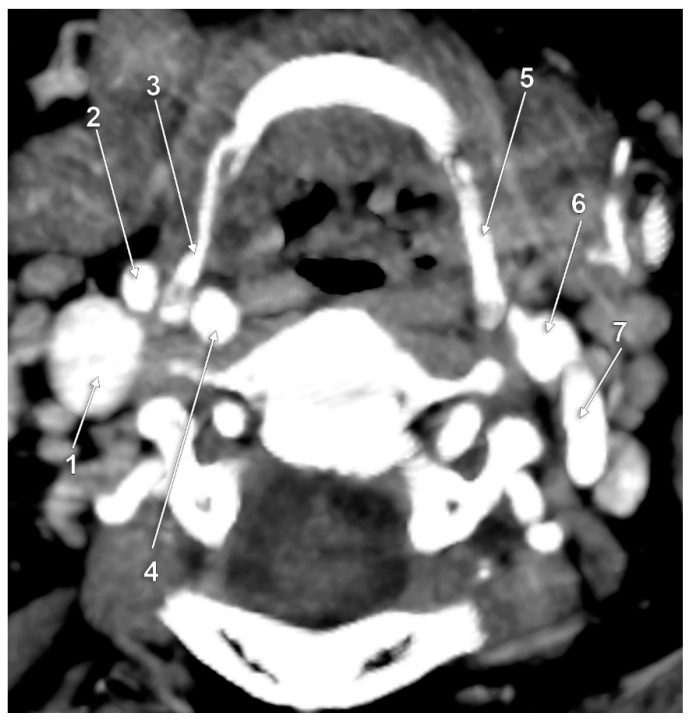
Right type XI of carotid–hyoid ratio. Axial slice, inferior view. 1: right internal jugular vein; 2: right external carotid artery; 3: right greater hyoid horn; 4: right internal carotid artery; 5: left greater hyoid horn; 6: left external carotid artery; 7: left internal carotid artery.

Type I (ECA medial to the GHHB) was evident in 0.34% (Figure 2), type II (ICA medial to the GHHB) was present in 0.34% (Figure 3), type III (ECA and ICA medial to the GHHB) was present in 1.02% (Figure 4), type IV (CCA medial to the GHHB) was present in 1.02% (Figure 5), type V (CB medial to the GHHB) in 0.34% (Figure 6), type VI (ECA lateral to the GHHB) was identified in 20.41% (Figure 7), type VIII (ECA and ICA lateral to the GHHB) in 3.74% (Figure 8), type IX (CCA lateral to the GHHB) in 8.5% (Figure 9), type X (CA lateral to the GHHB) in 6.46% (Figure 10), type XI (ICA medial and ECA lateral to the GHHB) in 0.34% (Figure 11), and type XII (ECA medial and ICA lateral to the GHHB) in 0.34% (Figure 4).

**Figure 12:**
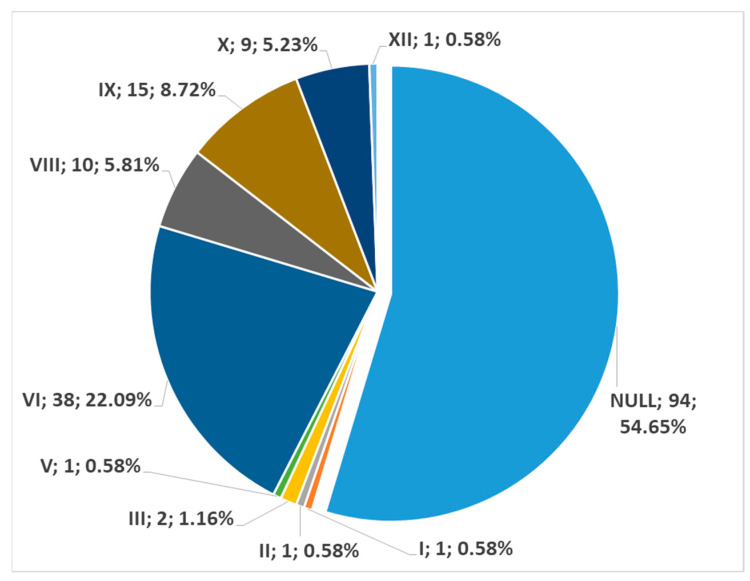
Distribution of carotid–hyoid relationship types in the male subgroup (172 sides). Type; count; prevalence.

**Figure 13:**
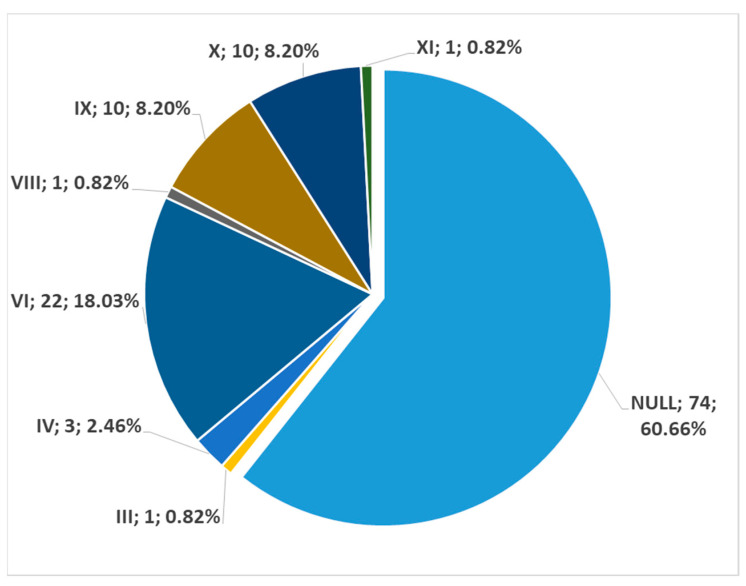
Distribution of carotid–hyoid relationship types in the female subgroup (122 sides). Type; count; prevalence.

**Figure 14:**
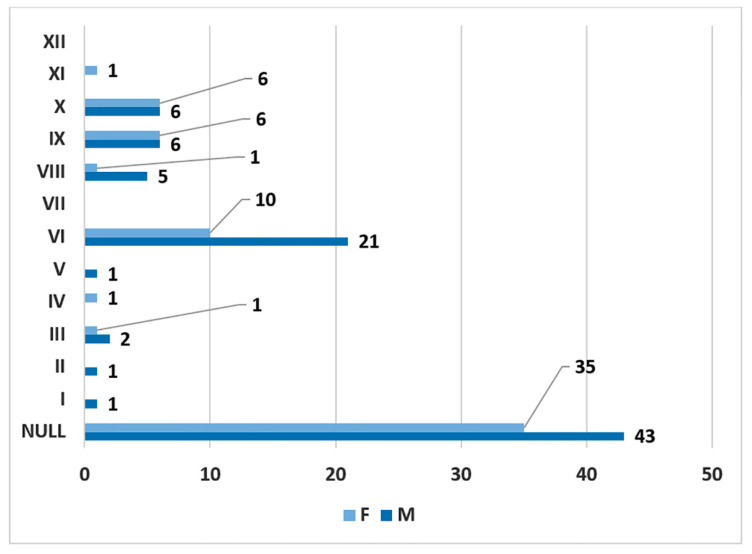
Gender distribution (F: female, M: male) of carotid–hyoid ratio types on the right side.

**Figure 15:**
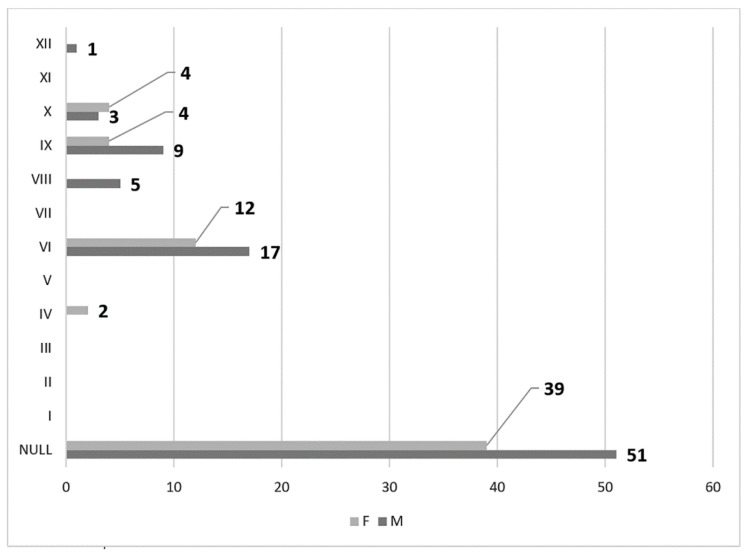
Gender distribution (F: female, M: male) of carotid–hyoid ratio types on the left side.

In men (172 sides), no carotid–hyoid relationships were found in 54.65% of individuals (Figure 12). We did not find any examples of types VII and XII in the male subgroup. In women (122 sides), we did not identify carotid–hyoid relationships in 60.66% of individuals (Figure 13). In the female subgroup, we did not find types I, II, III, V, VII, or XI. We determined the types of ratios comparatively by sex (Figure 14 and Figure 15).

**Figure 16:**
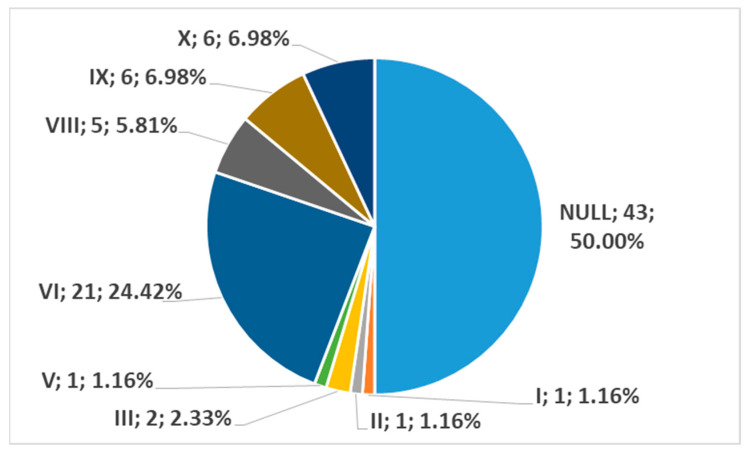
Distribution by specific type of carotid–hyoid relationships in males on the right side (n = 86).

**Figure 17:**
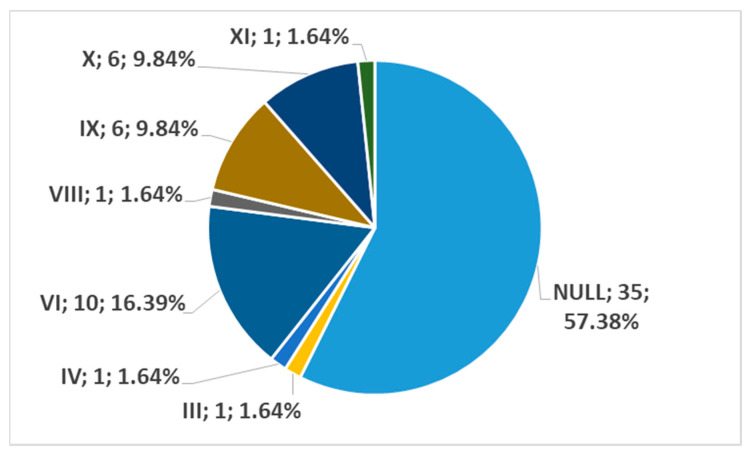
Distribution by specific type of carotid–hyoid relationships in females on the right side (n = 61).

In men, on the right side (n = 86), we found no carotid–hyoid ratios in 50% of individuals; type VI (ECA lateral to the GHHB) was found in 24.42% and types IX (CCA lateral to the GHHB) and X (CB lateral to the GHHB) were each present in 6.98% (Figure 16). In women, on the right side (n = 61), we found no carotid–hyoid relationships in 57.38% of individuals; type VI (ECA lateral to the GHHB) was identified in 16.39% and types IX (CCA lateral of hyoid) and X (CB lateral of hyoid) were each present in 9.84% (Figure 17).

**Figure 18:**
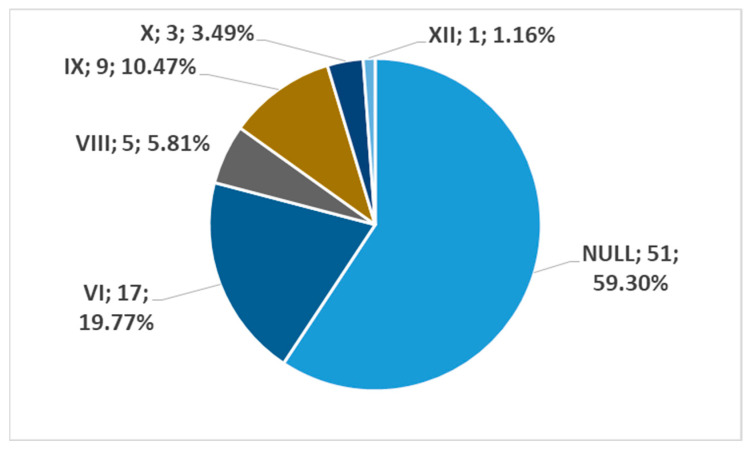
Distribution by specific type of carotid–hyoid relationships in men on the left side (n = 86).

**Figure 19:**
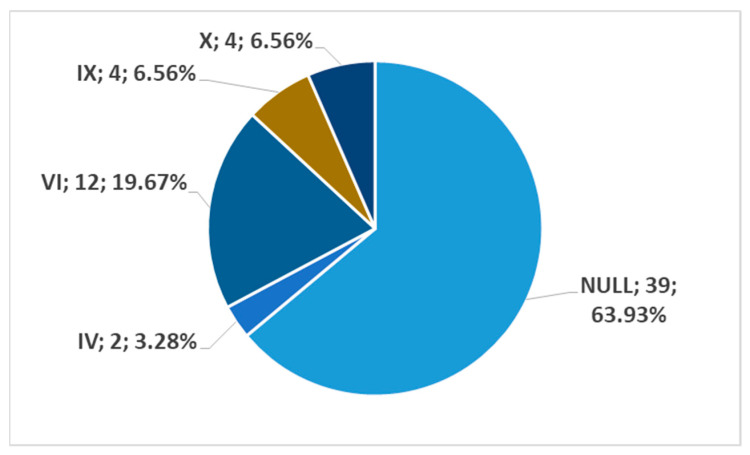
Distribution by specific type of carotid–hyoid relationships in women on the left side (n = 61).

In men, on the left side (n = 86), we found no carotid–hyoid relationships in 59.3% of individuals; type VI (ECA lateral to the greater hyoid horn) was identified in 19.77% and type IX (CCA lateral to the hyoid) was present in 10.47% (Figure 18). In females, on the left side (n = 61), we found no carotid–hyoid relationships in 63.93% of individuals; type VI (ECA lateral to the GHHB) was found in 19.67% and types IX (CCA lateral of hyoid) and X (CB lateral of hyoid) were each present in 6.56% (Figure 19).

**Figure 20:**
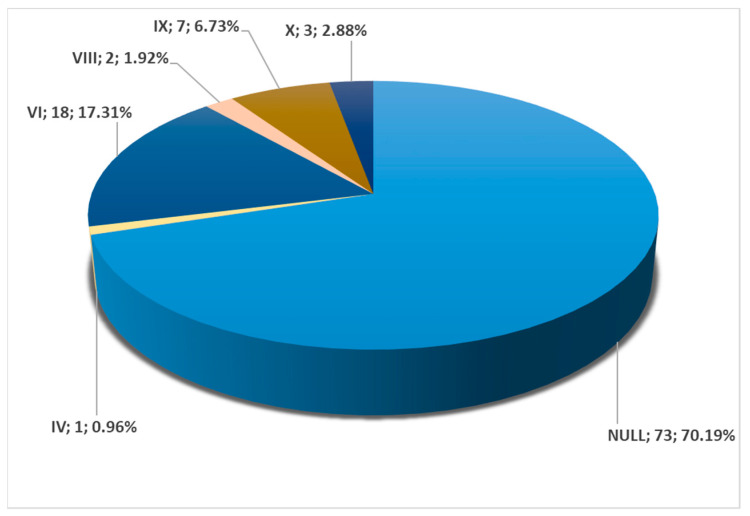
Bilateral symmetry of carotid–hyoid relationship types in the overall group (N = 147), including absent (null) and present.

Among all cases, 104/147 (70.74%) were found to have bilateral symmetry of the carotid–hyoid relationship. Of these 104 cases, 70.19% were null types without carotid–hyoid relationships, with type IV present in 0.96% (Figure 5), type VI in 17.31% (Figure 2), type VIII in 1.92% (Figure 3), type IX in 6.73% (Figure 4), and type X in 2.88% (Figure 5 and Figure 20). There was a highly significant association between the left and right variants of the carotid–hyoid relationship, with a Pearson Chi-squared value of 466,592, significant at a *p*-value below 0.001.

In men, we found 55/86 cases with bilateral symmetry; 39 of these showed no carotid-hyoid relationship, ten were type VI, two were type VIII, and four were type IX. In women there were 49/61 cases with bilateral carotid–hyoid ratio symmetry; 34 of these showed no carotid–hyoid relationship, one case was type IV, eight cases were type VI, three cases were type IX and three cases were type X.

## 4. Discussion

Bilateral symmetry was highly statistically significant and was identified in cases without carotid–hyoid relationships as well as in types IV, VI, VIII, IX, and X. Bilateral symmetry was not found for types I, II, III, V, VII, XI, and XII. Thus, these can be regarded as unilateral anatomical variations. In the present study, bilateral symmetry of the carotid–hyoid relationship was found in 70.74% of individuals. This is a topographic transverse symmetry of the carotid–hyoid relationship, and is not one of the vertical levels of CB. In a previous study, bilateral symmetry of the vertebral level of CB was found in just 28% of cases [16]. Bilateral symmetry for different morphofunctional characteristics of the carotid arteries was determined in youth, and significant differences were found only for the carotid lumen diameter [17].

However, it should be borne in mind that the hyoid bone has extremely heterogeneous morphological characteristics that are closely related to individual differences in sex, height, and weight [18]. Therefore, carotid variability is superposed over an individually variable hyoid morphology.

Lemaire et al. (2005) conducted a study on 30 cadavers to assess the relevance of the tip of the GHHB in the localization of the CB, superior thyroid and lingual arteries, and hypoglossal and superior laryngeal nerves [19]. The authors stated that they found no anatomical variant other than the level of origin of the superior thyroid artery [19]. The authors found that the CB is always located posterior and inferior to the tip of the GHHB [19]. These results support the importance of the present study, demonstrating that CB localization is not a consistent anatomical pattern.

Types with the ICA medial to the greater horn have rarely been reported [20,21,22,23]. Kolbel et al. (2008) reported one such case, with neurological symptoms that disappeared after resection of the respective greater hyoid horn [20]. In that case, the carotid–hyoid relationships were type XI on the right and type VI on the left [20]. Martinelli et al. (2019) identified a type XI carotid–hyoid relationship in one case [22]. A type XI relationship was found in one case by Liu et al. (2020), with the tip of the greater hyoid horn between the ECA (laterally) and ICA (medially) pressing on the carotid sinus or bulb [24]. Plotkin et al. (2019) reported a case with a type XI relationship [21]. Kho et al. (2019) reported a case with ICA and ECA medial to the greater horn, which is a type III relationship [23]. Tokunaga et al. (2015) presented evidence of a type II carotid–hyoid relationship [25].

Renard and Freitag (2012) reported a case in which the tip of the greater horn was located 1.6 mm anterior to the anterior tubercle of the transverse process of the C3 vertebra, while the ECA and ICA were located lateral to the greater horn, which is a type VIII relationship [26]. Mori et al. (2011) reported a case with the ICA and ECA lateral to the greater horn, which is a type VII relationship. It is anatomically interesting that a consistent linguofacial trunk arose from that ECA, ascending anterior to the ECA and lateral to the greater horn; thus, three arteries were located lateral to the greater horn [27]. Hong et al. (2011) identified the location of the ICA and ECA lateral to the greater horn in one case, which is a type VIII relationship [28]. A type VIII carotid–hyoid relationship was reported by Renard et al. (2011) [29]. Yukawa et al. (2014) reported a case having both ICA and ECA on one side lateral to the greater hyoid horn, which is a type VIII relationship, and the contralateral CB was lateral to the greater horn, which is a type X relationship [30]. A type X relationship with the CCA lateral to the greater horn was reported by Liu et al. (2021) [31]. Schneider and Kortmann (2007) reported a case with the location of the CCA lateral to the greater horn, which is a type IX relationship [32].

Feracci et al. (2022) quoted the study of Koreckij et al. (2013) regarding the three types of carotid vessel location as related to the spine: type I (normal), lateral to the transverse foramen; type II (medial), in the zone between the transverse foramen and the uncovertebral joint of Luschka; and type III (retropharyngeal), medial to the uncovertebral joint [33,34]. Koreckij et al. identified aberrant types II and III as abnormalities of the carotid arteries, but did not specify which carotid arteries these referred to [33]. The authors found types II and III in 12.3% of 1000 patients and that 26 cases (2.6%) had retropharyngeal type III [33]. The study by Koreckij et al. mainly addressed the anterior cervical spine approach (Smith–Robinson approach) for the treatment of radiculopathy and myelopathy, and recommended preoperative detection of the carotid tract in patients [33]. Anterior landmarks such as is the hyoid bone could be of better use during neck dissections, though this should be regarded with caution.

The hyoid bone is a remote cause of atherosclerotic lesions of the carotid arteries [22]. A retrospective cross-sectional and longitudinal cohort study, however, concluded that the presence and progression of atherosclerotic plaque and ICA stenosis do not depend on the distance between the hyoid and the ICA [35]. Mechanical arterial compression can lead to ICA dissection [26,30]. In addition, chronic ICA compression with repetitive vascular trauma can lead to cerebral thromboembolic strokes in young patients [21]. Compression of the CCA may evolve with perforation of its wall [36,37] and formation of a pseudoaneurysm of this artery [32]. As pseudoaneurysms can form in both the CCA and the ECA or ICA [38], mechanical compression by the hyoid can be considered and investigated in such cases. One case with mechanical compression of the ICA by the hyoid and frequent non-atherothrombotic occlusion and recanalization of the ICA was reported by [27]. Stenosis or occlusion of the ICA can occur directly by mechanical compression of the hyoid or indirectly by atheromatous plaque formation [29]. Hyoid fractures in cases positive for carotid–hyoid ratio can cause significant haemorrhage. Furthermore, hyoid fractures can cause pseudoaneurysms of the ECA [39].

Different types of carotid–hyoid relationships were found in this study. In types I–V, the CCA, BC, ECA, ICA, or both the ECA and ICA were found medially to the GHHB. These variants are of utmost importance during otorhinolaryngological surgeries such as tonsillectomy and drainage of peritonsillar abscesses, as well as during adenoidectomies and pharyngeal lesion biopsies [10,40]. Such anatomic variations should always be included in the differential diagnosis of pharyngeal wall bulging [40]. Specifically, surgeons should be aware that any carotid artery could be found medially to the GHHB, and as such posteriorly to the pyriform sinus (types I–V), which occurred in this study in 3.06% of individuals. The inadvertent performance of biopsies or punctures nearing the pyriform sinus could lead to fatal outcomes, especially because the parapharyngeal space is a difficult region to evaluate clinically due to its location deep in the neck [40].

The development of non-atherosclerotic or atherosclerotic ICA vasculopathy can be explained by repeated mechanical compression of the ICA between the sternocleidomastoid muscle and the greater horn of the hyoid bone [28]; this can result in types VI, VIII, IX, X, XII. When the head is rotated rapidly, the risk of injury tends to increase contralaterally [28,41]. Displacement of the origin of the ICA relative to the greater hyoid horn with head rotation and swallowing in daily life, causing repeated mechanical stimulation by the hyoid bone, could lead to endothelial damage, thrombus formation, and cerebral artery embolism [25].

When extrinsic carotid compression is evident, the endovascular treatment of carotid stenosis is not advisable and open surgery is preferred [22]. Good management for mechanical compression, however, has been achieved with endarterectomy and carotid mobilization without hyoid resection [24].

Displacement of ICA by the hyoid should be considered in strokes of unknown aetiology, especially in the presence of cervical, pharyngeal, or otic pain, repetitive cervical movements, and prolonged cervical positioning. In such cases, angioCT scanning with head rotation may be useful for screening [23].

## 5. Conclusions

Mechanical compression of the hyoid bone on the carotid arteries has various undesirable effects on the ICA and cerebral circulation. Underlying these are several variational anatomical patterns of carotid–hyoid relationships, which can be accurately documented on CT angiograms.

Based on the correlation between the variability of the carotid–hyoid relationship and individual hyoid variability, a case-by-case assessment of the carotid artery–hyoid bone relationships is preferable to preoperative assumption of a single possibility learned from anatomical textbooks and atlases.

Any cervical surgical approach involving intraoperative identification of the hyoid bone should be done with caution, as it cannot be excluded that this bone might have a direct and immediate relationship with the carotid arteries either laterally or medially.

## Data Availability

No new data were created or analyzed in this study. Data sharing is not applicable to this article.
